# Multilocus pathogenic variants contribute to intrafamilial clinical heterogeneity: a retrospective study of sibling pairs with neurodevelopmental disorders

**DOI:** 10.1186/s12920-024-01852-4

**Published:** 2024-04-16

**Authors:** Tugce Bozkurt-Yozgatli, Davut Pehlivan, Richard A. Gibbs, Ugur Sezerman, Jennifer E. Posey, James R. Lupski, Zeynep Coban-Akdemir

**Affiliations:** 1 Department of Biostatistics and Bioinformatics, Institute of Health Sciences, Acibadem Mehmet Ali Aydinlar University, Istanbul, Turkey; 2 Human Genetics Center, Department of Epidemiology, Human Genetics, and Environmental Sciences, School of Public Health, University of Texas Health Science Center at Houston, Houston, TX USA; 3 Department of Pediatrics, Baylor College of Medicine, Houston, TX USA; 4 Section of Pediatric Neurology and Developmental Neuroscience, Department of Pediatrics, Baylor College of Medicine, Houston, TX USA; 5 Jan and Dan Duncan Neurological Research Institute at Texas Children’s Hospital, Houston, TX USA; 6 Texas Children’s Hospital, Houston, TX USA; 7 Department of Molecular and Human Genetics, Baylor College of Medicine, Houston, TX 77030 USA; 8 Human Genome Sequencing Center, Baylor College of Medicine, Houston, TX USA; 9 Department of Biostatistics and Medical Informatics, School of Medicine, Acibadem Mehmet Ali Aydinlar University, Istanbul, Turkey

**Keywords:** Exome sequencing, Runs-of-homozygosity, Multilocus pathogenic variation, Neurodevelopmental disorders

## Abstract

**Background:**

Multilocus pathogenic variants (MPVs) are genetic changes that affect multiple gene loci or regions of the genome, collectively leading to multiple molecular diagnoses. MPVs may also contribute to intrafamilial phenotypic variability between affected individuals within a nuclear family. In this study, we aim to gain further insights into the influence of MPVs on a disease manifestation in individual research subjects and explore the complexities of the human genome within a familial context.

**Methods:**

We conducted a systematic reanalysis of exome sequencing data and runs of homozygosity (ROH) regions of 47 sibling pairs previously diagnosed with various neurodevelopmental disorders (NDD).

**Results:**

We found siblings with MPVs driven by long ROH regions in 8.5% of families (4/47). The patients with MPVs exhibited significantly higher F_ROH_ values (*p*-value = 1.4e-2) and larger total ROH length (*p*-value = 1.8e-2). Long ROH regions mainly contribute to this pattern; the siblings with MPVs have a larger total size of long ROH regions than their siblings in all families (*p*-value = 6.9e-3). Whereas the short ROH regions in the siblings with MPVs are lower in total size compared to their sibling pairs with single locus pathogenic variants (*p*-value = 0.029), and there are no statistically significant differences in medium ROH regions between sibling pairs (*p*-value = 0.52).

**Conclusion:**

This study sheds light on the significance of considering MPVs in families with affected sibling pairs and the role of ROH as an adjuvant tool in explaining clinical variability within families. Identifying individuals carrying MPVs may have implications for disease management, identification of possible disease risks to different family members, genetic counseling and exploring personalized treatment approaches.

**Supplementary Information:**

The online version contains supplementary material available at 10.1186/s12920-024-01852-4.

## Background

Next-generation sequencing (NGS) technologies and advanced bioinformatics tools have enabled the detection of genetic variants by simultaneously sequencing multiple genes. These advancements facilitate the increasing recognition of multilocus pathogenic variants (MPVs) [1–6]. MPVs refer to genetic alterations that occur in multiple genes or genomic regions [7]. Unlike phenotypic expansions of a well-characterized monogenic condition, MPVs may result in multiple molecular diagnoses that drive blended phenotypes. The frequency of reported multiple molecular diagnoses ranges from 2.4 to 7.2% in different studies [1, 5, 8]. The identification of MPVs can facilitate an improved understanding of personalized treatment approaches and estimates of familial recurrence risk.

ROH regions are consecutive blocks of homozygous genotypes, which arise from inheriting two identical haplotypes, one from each parent. Analyzing the burden and size of ROH regions across entire individual genomes provides valuable insights into the demographic and evolutionary history of a population or a nuclear family [9, 10]. Clark et al. showed that ROH burden is linked to several human phenotypes including reduced reproductive success, reduced risk-taking behavior, and increased disease risk [11]. They also provide results of within-siblings approach, showing that siblings with higher F_ROH_ (fraction of the genome covered by ROH regions > 1.5 Mb) experience poorer overall health compared to their siblings [11]*.* Additionally, Gamsiz et al. showed increased ROH burden in children with autism spectrum disorders compared to their unaffected siblings [10].

Based on the size classification in Pemberton et al., short and medium ROH regions may be influenced by ‘locus-aware’ homozygosity and linkage disequilibrium; long ROH regions are more likely to have been formed due to recent inbreeding [12]. Several previous studies have demonstrated the enrichment of deleterious variants in long ROH regions [13, 14]. This phenomenon has been hypothesized to result from the absence of sufficient generational time for the selective removal of long/young haplotypes containing rare deleterious variants from the population [15, 16]. Furthermore, several prior studies have shown that ROH burden in an individual genome drives MPVs [1, 3, 4]. However, these studies focused on the total ROH size of unrelated individuals without any ROH size classification. We present a systematic reanalysis of exome sequencing data (ES) and classification of ROH regions from 47 sibling pairs previously diagnosed with neurodevelopmental disorders (NDD). Our objective is to delve deeper into the impact of MPVs on the manifestation of disease phenotypes within a nuclear family and acquire an additional understanding of the current literature regarding how ROH classes influence familial clinical heterogeneity.

## Methods

### Participants

Here, we performed a retrospective analysis of 47 sibling pairs with NDDs in the Baylor Hopkins Center for Mendelian Genomics (BHCMG) cohort, for whom the variants explanatory for their phenotypes have been reported by Karaca et al. [17] and Mitani et al. [4].

### Variant analysis

We investigated homozygous, heterozygous, and compound-heterozygous variant alleles. Rare variants (< 0.01%) were prioritized according to frequency in population databases, including the 1000 Genomes Project, gnomAD, and our in-house-generated exome database (personal genome exomes from ∼13,000 individuals) at the Baylor College of Medicine Human Genome Sequencing Center (BCM-HGSC). Rare variants with a Combined Annotation Dependent Depletion (CADD)-Phred score of > 15 were retained. Candidate SNVs that remained after the ES analysis were orthogonally validated (Sanger dideoxy sequencing) and segregated in available family members via an orthogonal approach. Beyond examining patient-specific variants, we also assessed variants shared among sibling pairs. After this filtering step, we next focused on pathogenic, likely pathogenic variants based on the American College of Medical Genetics and Genomics (ACMG) criteria [18]. However, we further evaluated variants of uncertain significance to seek additional supporting evidence for a likelihood of pathogenicity.

### Runs of homozygosity (ROH) analysis

We detected ROH regions from ES data using BafCalculator as described [14]. We classified each ROH genomic interval into three size categories (short, medium, and long) based on the previously defined size cut-offs for the Turkish population, which are 0.210–0.671 Mb for short, 0.671–2.64 Mb for medium, and > 2.64 Mb for long ROH regions [14]. We also calculated the fraction of the genome covered by ROH regions > 1.5 Mb (F_ROH_).

### Statistical analysis

All statistical analyses were performed in R (v.4.2.0) [19]. The plots given in the study were generated by the ggplot2 (https://github.com/tidyverse/ggplot2) and ggpubr R packages (https://github.com/kassambara/ggpubr).

### Sanger sequencing

Sanger sequencing was conducted on the samples with an MPV following the previously reported protocol [4]. The primer pairs used in this study are provided in the supplementary material.

## Results

We reanalyzed ES data from 47 families with two affected sibling pairs with NDDs (Supplementary Fig. 1). In addition to their initial molecular diagnosis, we reported variant alleles from another locus that may be clinically relevant in one of the siblings in each of the 4 families (Table 1).

**Table 1:** Genotype and phenotype information in 4 families with a sibling with MPVs

Pedigree ID	Individual ID	Clinical phenotype of the patients	Initially identified variants / Zygosity	gnomAD AF (Exomes/Genomes)	Additional variants identified in this study / Zygosity	gnomAD AF (Exomes/Genomes)	OMIM Phenotype	ACMG classification of the additional variants	Other Evidence
HOU1842	BAB4133	microcephaly, developmental delay, and intellectual disability	*TNN*:NM_022093:c.2516G > A:p.R839K / homozygous	0/0					
	BAB4134				*CYP1B1:*NM_000104.4:c.182G > A:p.G61E / homozygous	0.000318/ 0.000128	Anterior segment dysgenesis 6, multiple subtypes (OMIM #617315) Glaucoma 3A, primary open angle, congenital, juvenile, or adult onset (OMIM #231300)	Pathogenic	Bejjani et al.*,* AJHG,1998;Stoilov et al.*,* AJHG, 1998
HOU2280	BAB6025	Neurodevelopmental disorders	*ASH2L*:NM_001105214:c.1444A > G: p.I482V / homozygous	0/0	*ECEL1:*NM_004826.4:c.2012G > A:p.G671E / homozygous	0/0	Arthrogryposis type-5D (OMIM #615065)	Likely Pathogenic	
	BAB6026								
HOU2437	BAB6511	developmental delay, intellectual disability, microcephaly, and epilepsy	*CINP*:NM_032630:c.637 T > G:p.*213Gext*21 / homozygous	0/0	*MFN2:*NM_014874.4:c.1555C > T: p.R519C / homozygous	0.0000159/0	Charcot-Marie-Tooth disease type 2A2B (OMIM #617087) Lipomatosis, multiple symmetric, with or without peripheral neuropathy (OMIM #151800).	Uncertain Significance	Likely pathogenic (ClinVar Variation ID: 522942)
	BAB6512								
HOU4131	BAB11385	spasticity, increased deep tendon reflexes, hirsutism, intellectual disability and neuromotor delay	*ASXL3*:NM_030632.3:c.2213C > T:p.S738F / homozygous	0/0					
	BAB11388				*PLA2G6:*NM_003560.4:c.16C > T:p.R6C / homozygous	0.000119/ 0.000127	Infantile neuroaxonal dystrophy 1 (OMIM #256600) Neurodegeneration with brain iron accumulation 2B (OMIM #610217) Parkinson disease 14, autosomal recessive (OMIM #612953)	Uncertain Significance	Mahdieh et al., Sci Rep. 2021

### Family 1

**Fig. 1:**
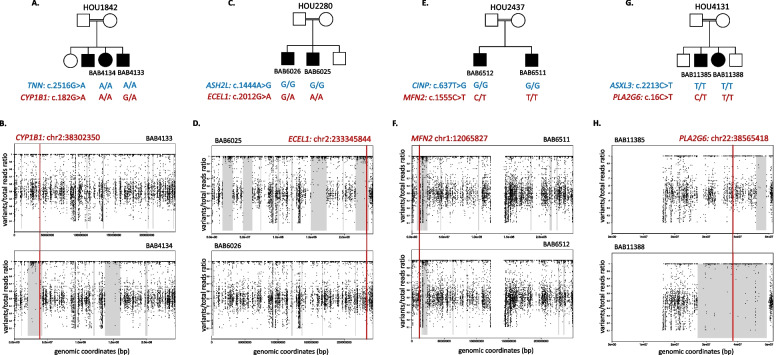
Pedigree and ROH plots of the families with a sibling with MPVs. **A** Pedigree structure and molecular findings in family HOU1842. Both affected siblings share a homozygous variant in *TNN*; BAB4133 has additional homozygous variants in *CYP1B1*. **B** ROH plot around the *CYP1B1* variant in BAB4133 and BAB4134. B-allele frequency calculated from ES data is visualized as horizontal black dots. ROH blocks are marked by gray rectangles. Red vertical line marks the *CYP1B1* variant position. **C** Pedigree structure and molecular findings in HOU2280. Both affected siblings share a homozygous variant in *ASH2L*; BAB6025 has additional homozygous variants in *ECEL1*. **D** ROH plot around the *ECEL1* variant in BAB6025 and BAB6026. B-allele frequency calculated from ES data is visualized as horizontal black dots. ROH blocks are marked by gray rectangles. Red vertical line marks the *ECEL1* variant position. **E** Pedigree structure and molecular findings in HOU2437. Both affected siblings share a homozygous variant in *CINP*; BAB4133 has additional homozygous variants in *MFN2*. **F** ROH plot around the *MFN2* variant in BAB6511 and BAB6512. B-allele frequency calculated from ES data is visualized as horizontal black dots. ROH blocks are marked by gray rectangles. Red vertical line marks the *MFN2* variant position. **G** Pedigree structure and molecular findings in HOU4131. **H** ROH plot around the *PLA2G6* variant in BAB11385 and BAB11388. B-allele frequency calculated from ES data is visualized as horizontal black dots. ROH blocks are marked by gray rectangles. Red vertical line marks the *PLA2G6* variant position

In family HOU1842 with reported 1st-degree cousin parents (Fig. 1A), the initial ES analysis demonstrated a molecular diagnosis of microcephaly, developmental delay (DD), and intellectual disability (ID) due to a homozygous pathogenic variant in *TNN* (HGNC:22942) in both siblings (Supplementary Fig. 2B) [17]. This study revealed a pathogenic (PP5_Very Strong, PM5_Strong, PM1_Moderate, PP3_Moderate) variant (NM_000104.4:c.182G > A p.Gly61Glu) in *CYP1B1* (HGNC:2597) in BAB4134 (Fig. 1B). The frequency of the *CYP1B1* variant allele is 0.000318 and 0.000128, and 0.000971 in the gnomAD [20] exome, genome, and our in-house exome database, respectively. The Gly61Glu variant was also reported in a Turkish patient with primary congenital glaucoma [21]. The variant is within a long ROH with 18.06 Mb length in BAB4134 (Fig. 1B). Pathogenic homozygous CYP1B1 (HGNC:2597) variants are associated with Anterior segment dysgenesis 6, multiple subtypes (OMIM #617315) and Glaucoma 3A, primary open angle, congenital, juvenile, or adult onset (OMIM #231300).

### Family 2

In family HOU2280 (Fig. 1C), the initial ES analysis detected a homozygous variant in *ASH2L* (HGNC:744) in both siblings with NDD, who were born into a family reported with an unknown degree of consanguinity (Supplementary Fig. 3B) [17]. Here, we identified the novel variant (NM_004826.4:c.2012G > A p.Gly671Glu) in *ECEL1* (HGNC:3147) classified as likely pathogenic (PP3_Strong, PM1_Supporting, PM2_Supporting) based on the ACMG criteria in BAB6025 (Fig. 1D). The *ECEL1 variant* is not found in the gnomAD [20] (allele frequency (AF) is 0 in both the exome and genome datasets). However, the AF of the variant is 0.000092 in our in-house exome database. The *ECEL1* (HGNC:3147) variant is within a 19.42 Mb-length long ROH in BAB6025 (Fig. 1D). Homozygous pathogenic variants in *ECEL1* (HGNC:3147) are associated with Arthrogryposis type-5D (OMIM # 615065).

### Family 3

In family HOU2437 with reported 1st-degree cousin parents (Fig. 1E), the pathogenic variant in *CINP* (HGNC:23789) was initially reported in both siblings with phenotypes of DD, ID, microcephaly, and epilepsy (Supplementary Fig. 4B) [17]. In addition to this variant, BAB6511 has the novel variant (NM_014874.4:c.1555C > T p.Arg519Cys) in the *MFN2* (HGNC:16877) gene (Fig. 1F). The variant is classified as of uncertain significance (PM5_Moderate, PM2_Supporting, PP2_Supporting, PP5_Supporting, BP4_Supporting) based on the variant classifications recommended by the ACMG [18], while it is reported to be likely pathogenic in ClinVar (Variation ID: 522942). The AF of the *MFN2* variant is 0.0000159, 0, and 0.000092 in the gnomAD [20] exome, genome, and our in-house exome database, respectively. Also, this variant is within a 5.31 Mb-length long ROH in BAB6511 (Fig. 1F). Homozygous pathogenic variants in *MFN2* (HGNC:16877) are associated with Charcot-Marie-Tooth disease type 2A2B (OMIM #617087) and Lipomatosis, multiple symmetric, with or without peripheral neuropathy (OMIM #151800).

### Family 4

In family HOU4131 (Fig. 1G), previous analyses revealed a pathogenic variant in *ASXL3* (HGNC:29357) in both siblings with spasticity, increased deep tendon reflexes, hirsutism, ID, and neuromotor delay (Supplementary Fig. 5B) who were born into a family reported with an unknown degree of consanguinity [4]. Here, we detected the variant (NM_003560.4:c.16C > T p.Arg6Cys) in *PLA2G6* (HGNC:9039) in BAB11388 (Fig. 1H). This variant is classified as one of uncertain significance (i.e.*,* VUS; PM2_Supporting, PP2_Supporting, BP4_Supporting); however, it was previously reported in a patient with hypotonia, bristled hair, and seizure [22]. The frequency of the *PLA2G6* variant allele is 0.000119, 0.000127, and 0.00069 in the gnomAD [20] exome, genome, and our in-house exome database, respectively. The variant maps within an 18.44 Mb ROH in BAB11388. (Fig. 1H). Homozygous pathogenic *PLA2G6* variants are the causes of Infantile neuroaxonal dystrophy 1 (OMIM #256600), Neurodegeneration with brain iron accumulation 2B (OMIM #610217), and Parkinson disease 14 (OMIM #612953).

**Fig. 2:**
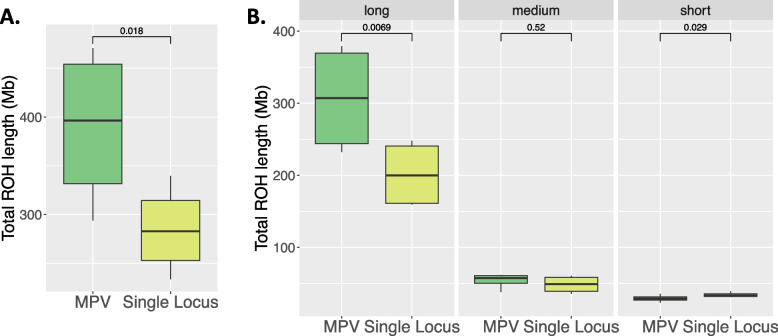
Statistical analyses of ROH patterns in the siblings. **A** Box plot of total ROH lengths in siblings with MPV vs. siblings with single locus pathogenic variant. **B** Box plot of total ROH lengths in siblings with MPV vs. siblings with single locus pathogenic variant for each ROH size category (long, medium, and short)

We also retrospectively analyzed previously detected causative variants in the sibling pairs. 83% of previously detected variants are within a long ROH region (Supplementary Fig. 6). Moreover, all additional variants identified in this study reside within long ROH regions. We found that the siblings with MPVs exhibited a significantly larger total size of ROH (Paired t-test, *p*-value = 1.8e-2, Fig. 2A). Consistent with this finding, the average F_ROH_ values are significantly higher in siblings with MPVs compared to their siblings with single locus variants (Supplementary Fig. 7, *p*-value = 1.4e-2). We also showed that siblings with MPVs exhibited significantly larger total size of long ROH regions in all families (Paired t-test, *p*-value = 6.9e-3, Fig. 2B). On the contrary, siblings with single locus variants have significantly larger total size of short ROH regions in all families (Paired t-test, *p*-value = 2.9e-2, Fig. 2B). On the other hand, there was no statistically significant difference in the total size of medium ROH regions between sibling groups (Paired t-test, *p*-value = 5.2e-1, Fig. 2B). The total size of short, medium, and long ROH sizes and F_ROH_ values in each individual are provided in Supplementary Table 1.

We also retrospectively analyzed previously detected causative variants in the sibling pairs. 83% of previously detected variants are within a long ROH region (Supplementary Fig. 6). Moreover, all additional variants identified in this study reside within long ROH regions. We found that the siblings with MPVs exhibited a significantly larger total size of ROH (Paired t-test, *p*-value = 1.8e-2, Fig. 2A). Consistent with this finding, the average F_ROH_ values are significantly higher in siblings with MPVs compared to their siblings with single locus variants (Supplementary Fig. 7, *p*-value = 1.4e-2). We also showed that siblings with MPVs exhibited significantly larger total size of long ROH regions in all families (Paired t-test, *p*-value = 6.9e-3, Fig. 2B). On the contrary, siblings with single locus variants have significantly larger total size of short ROH regions in all families (Paired t-test, *p*-value = 2.9e-2, Fig. 2B). On the other hand, there was no statistically significant difference in the total size of medium ROH regions between sibling groups (Paired t-test, *p*-value = 5.2e-1, Fig. 2B). The total size of short, medium, and long ROH sizes and F_ROH_ values in each individual are provided in Supplementary Table 1.

## Discussion

In this study, we investigated the role of MPVs in intrafamilial phenotypic variability in 47 sibling pairs recruited from the Turkish population. We used the term “phenotypic variability” to describe the clinical distinctions observed between two sibling pairs in the study. It’s important to note that these differences extend beyond the clinical spectrum of NDD; we used this expression in a more comprehensive context. 76.6% of the sibling pairs (36/47) were reported to be born to a consanguineous family by historical report. We reanalyzed ES data and ROH regions and identified siblings with MPVs driven by long ROH regions in 8.5% of families (4/47). In family HOU1842, the additional pathogenic variant in *CYP1B1* (HGNC:2597) [21] was identified in BAB4134, who was initially diagnosed with microcephaly and DD and ID. Affected siblings were severely delayed with no communication skills and died at young ages. By parental report, BAB4134 did not show any signs of eye disturbance such as rubbing or pointing to the eyes. Since the subject was deceased, we could not perform an ophthalmologic evaluation to objectively assess for glaucoma or anterior segment dysgenesis. In family HOU2280, in addition to the initially reported *ASH2L* (HGNC:744) variant, we identified a novel variant in *ECEL1* (HGNC:3147) classified as likely pathogenic based on the ACMG criteria in BAB6025. We were unable to contact the family to conduct an objective assessment of *ECEL1*-related findings in the patient. In family HOU2437, in addition to the initially detected *CINP* (HGNC:23789) variant contributing to the NDD phenotype characterized by DD, ID, microcephaly, and epilepsy, BAB6511 has a homozygous variant in *MFN2* (HGNC:16877) which was reported to be likely pathogenic in ClinVar (Variation ID: 522942). However, we could not reach out to the patient to objectively assess the *MFN2*-related findings. Although we could not evaluate family HOU2280 and HOU2437, we still report the variants identified based on solid in-silico evidence for the pathogenicity. In family HOU4131, both siblings were born at term after an uncomplicated pregnancy and delivery. Both had increased deep tendon reflexes, spasticity, hirsutism, and severe neurodevelopmental delay. BAB11388, who carries the *PLA2G6* variant is more severely affected, i.e., she did not achieve ambulation while BAB11385 was walking, and she said single words at 4 years, whereas her brother said single words at 3 years old (both siblings only had single words). Although we did not have brain imaging findings to assess whether there is ‘eye of the tiger sign’ on brain MRI, the patient’s physical exam findings, including severe DD/ID, spasticity, and increased deep tendon reflexes overlap with neurodegeneration with brain iron accumulation 2B. The more severe phenotype in BAB11388 is likely attributable to the deleterious variant in *PLA2G6* (HGNC:9039).

Our ROH findings are consistent with the previous studies [1, 3, 4], total ROH length is significantly larger in the patients with MPVs (Paired t-test, *p*-value = 1.8e-2, Fig. 2A). Besides, F_ROH_ values on average are significantly higher in the siblings with MPVs compared to the siblings with single locus variant, respectively (*p*-value = 1.4e-2, Supplementary Fig. 7). We also examined the ROH size categories contributing to this pattern between sibling groups. The siblings with MPVs have significantly larger total size of long ROH regions in all families (Paired t-test, *p*-value = 6.9e-3, Fig. 2B). In contrast to long ROHs, the short ROH regions in the siblings with MPVs are lower in total size compared to their sibling pairs with single locus pathogenic variants (Paired t-test, *p*-value = 0.029, Fig. 2B). On the other hand, there are no statistically significant differences in medium ROH regions between sibling pairs (Paired t-test, *p*-value = 0.52, Fig. 2B). Based on the findings in our study, we propose family members with larger size of long ROH regions should have their personal genome data carefully evaluated for a potential second locus contributing variant allele, particularly when intrafamilial phenotypic variability is observed.

Lastly, it is crucial to keep in mind the complexity of the human genome. Other biological factors such as variable expressivity of variants [23], epigenetic mechanisms [24] may play a role in contributing to phenotypic differences between sibling pairs. Additionally, the presence of different VUS without any current literature evidence in one of the sibling pairs may cause phenotypic variability between sibling pairs. Here, we present an insight into intrafamilial clinical variability from an ROH-driven MPVs perspective under the current knowledge of genetics literature.

## Conclusions

MPVs may exist in families with affected sibling pairs, and especially long ROH regions can be utilized as an adjuvant tool to uncover an MPV, wherein the second locus parsimoniously explains intra-familial phenotypic differences. Ultimately, this study sheds light on the significance of considering MPVs and the role of ROHs in explaining phenotypic variability within families, which includes individuals affected by rare disorders. Furthermore, the identification of individuals carrying MPVs can aid in genetic counseling, enabling more accurate risk assessment for disease prognosis and interventions and searching for personalized treatment strategies.

### Supplementary Information

**Additional file 1: Supplementary Fig. 1.** Overview of the study. Flow chart depicting study design. We performed a retrospective analysis of 47 sibling pairs diagnosed with neurodevelopmental disorders (NDDs) within the Baylor Hopkins Center for Mendelian Genomics (BHCMG) cohort. The variants responsible for their phenotypes were previously documented by Karaca et al. (*n* = 24) [17] and Mitani et al. (*n* = 23) [4].

**Additional file 2: Supplementary Fig. 2.** Pedigree and ROH plots of family HOU1842. A) Pedigree structure and molecular findings in HOU1842. B) B-allele frequency calculated from ES data is visualized as horizontal black dots. ROH blocks are marked by gray rectangles. The shared *TNN* variant in BAB4133 and BAB4134 is in an ROH region; the blue vertical line marks the position of the variant allele.

**Additional file 3: Supplementary Fig. 3.** Pedigree and ROH plots of family HOU2280. A) Pedigree structure and molecular findings in HOU2280. B) B-allele frequency calculated from ES data is visualized as horizontal black dots. ROH blocks are marked by gray rectangles. The shared *ASH2L* variant in BAB6025 and BAB6026 is in an ROH region; the blue vertical line marks map the position of the variant allele.

**Additional file 4: Supplementary Fig. 4.** Pedigree and ROH plots of family HOU2437. A) Pedigree structure and molecular findings in HOU2437. B) B-allele frequency calculated from ES data is visualized as horizontal black dots. ROH blocks are marked by gray rectangles. The shared *CINP* variant in BAB6511 and BAB6512 is in an ROH region; the blue vertical line marks map the position of the variant allele.

**Additional file 5: Supplementary Fig. 5.** Pedigree and ROH plots of family HOU4131. A) Pedigree structure and molecular findings in HOU4131. B) B-allele frequency calculated from ES data is visualized as horizontal black dots. ROH blocks are marked by gray rectangles. The shared *ASXL3* variant in BAB11385 and BAB11388 is within an ROH region; the blue vertical line marks map the position of the variant allele.

**Additional file 6: Supplementary Fig. 6.** Percentage of ROH size categories covering previously identified pathogenic variants in the patients. 83% of previously detected variants are within a long ROH (light blue); whereas 8.5% of them reside in a medium ROH (light green) and non-ROH (light yellow). It is noteworthy that 76.6% of the sibling pairs (36/47) were reported to be born to a consanguineous family by historical report.

**Additional file 7: Supplementary Fig. 7.** Box plot of F_ROH_ in siblings with MPV vs. siblings with single locus pathogenic variant. The average FROH values are notably higher in siblings with multiple pathogenic variants (MPVs) when compared to those with a single locus variant (*p*-value = 1.4e-2). The gray lines connect the FROH value data points for each sibling pair.

**Additional file 8: Supplementary Table 1.** The total size of short, medium, and long ROH regions and F_ROH_ values in each individual.

**Additional file 9: Supplementary file.** The primer sequences used in the orthogonal confirmation.

## Data Availability

For additional details regarding data and genomic analyses, please contact James R. Lupski (jlupski@bcm.edu), one of the corresponding authors.
