# Correction: Multilocus pathogenic variants contribute to intrafamilial clinical heterogeneity: a retrospective study of sibling pairs with neurodevelopmental disorders

**DOI:** 10.1186/s12920-024-01968-7

**Published:** 2024-08-07

**Authors:** Tugce Bozkurt‑Yozgatli, Davut Pehlivan, Richard A. Gibbs, Ugur Sezerman, Jennifer E. Posey, James R. Lupski, Zeynep Coban‑Akdemir

**Affiliations:** 1 Department of Biostatistics and Bioinformatics, Institute of Health Sciences, Acibadem Mehmet Ali Aydinlar University, Istanbul, Turkey; 2 Human Genetics Center, Department of Epidemiology, Human Genetics, and Environmental Sciences, School of Public Health, University of Texas Health Science Center at Houston, Houston, TX USA; 3 Department of Pediatrics, Baylor College of Medicine, Houston, TX USA; 4 Section of Pediatric Neurology and Develop- Mental Neuroscience, Department of Pediatrics, Baylor College of Medicine, Houston, TX USA; 5 Jan and Dan Duncan Neurological Research Institute at Texas Children’s Hospital, Houston, TX USA; 6 Texas Children’s Hospital, Houston, TX USA; 7 Department of Molecular and Human Genetics, Baylor College of Medicine, Houston, TX 77030 USA; 8 Human Genome Sequencing Center, Baylor College of Medicine, Houston, TX USA; 9 Department of Biostatistics and Medical Informatics, School of Medicine, Acibadem Mehmet Ali Aydinlar University, Istanbul, Turkey

**Correction** **:** **BMC Med Genomics 17, 85 (2024)**

**https://doi.org/10.1186/s12920-024-01852-4**

Following the publication of the Original Article [1], the authors reported an error in the placement of the plots during the manuscript preparation step. The authors accidentally placed the plots for Family HOU1842 in the Family of HOU4131, which resulted the duplication of Figure 1 Plots B and H and Supplementary Figure 2B and 5B.

**Incorrect**

**Figure** **1**


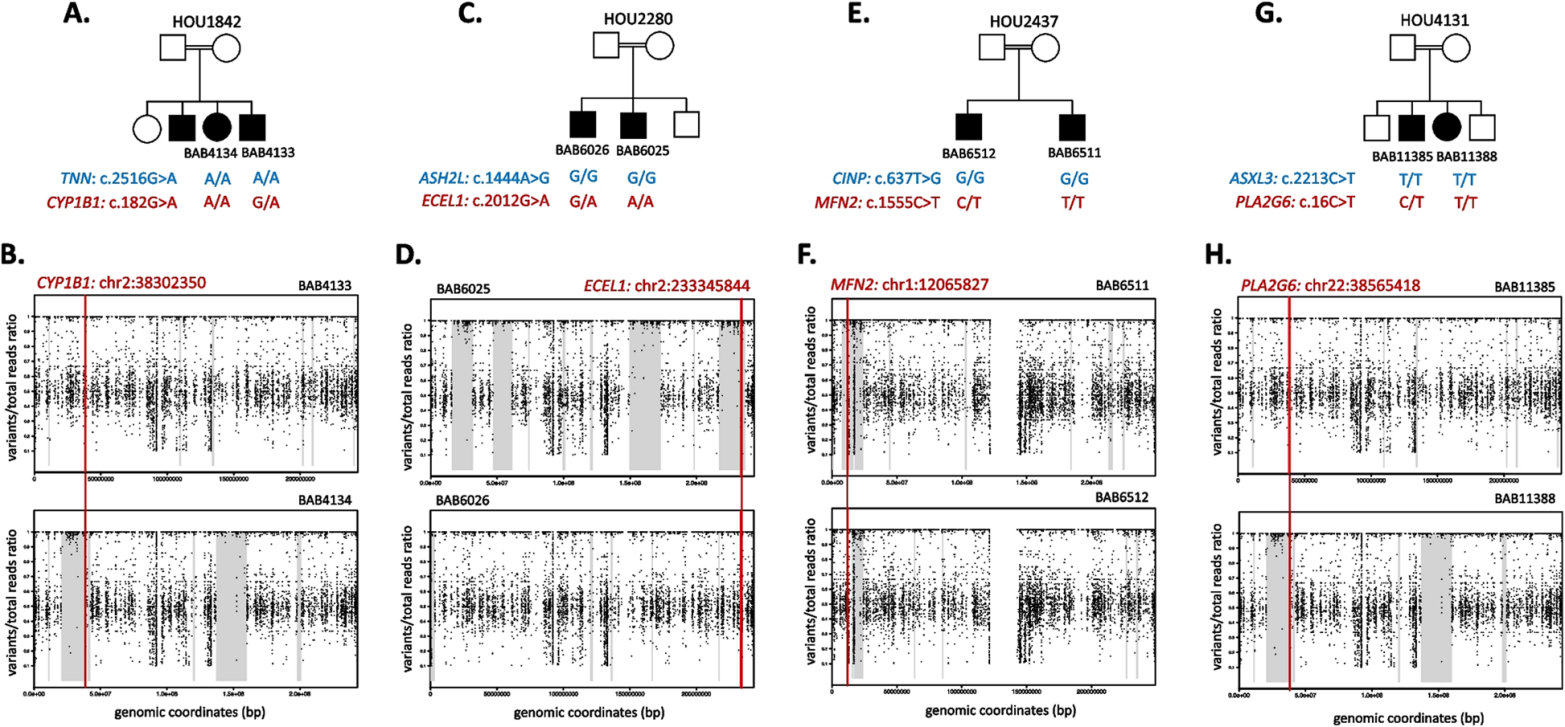


**Supplementary Figure** **5**


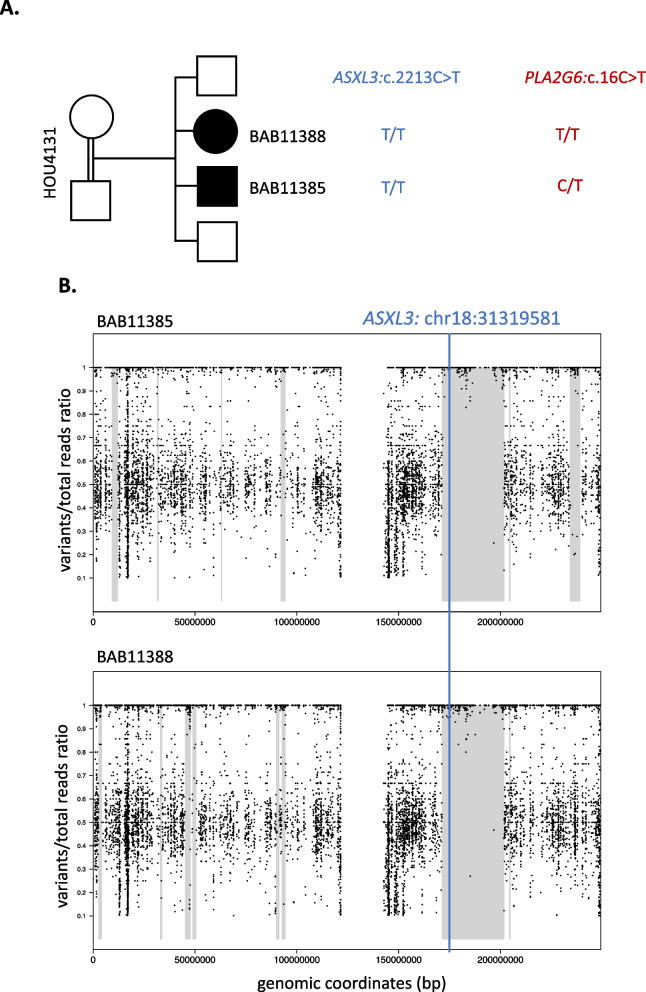


**Correct**

**Figure** **1**


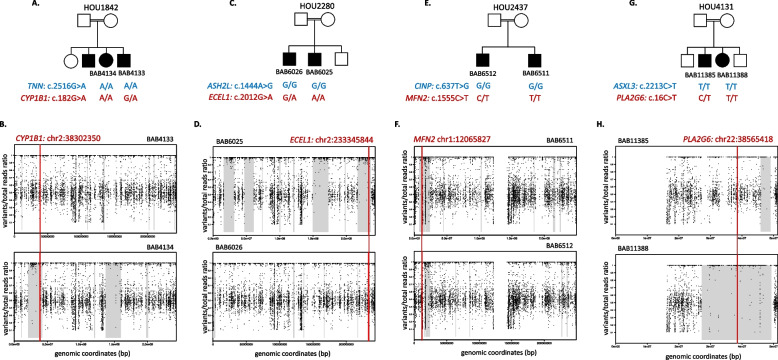


**Supplementary Figure** **5**


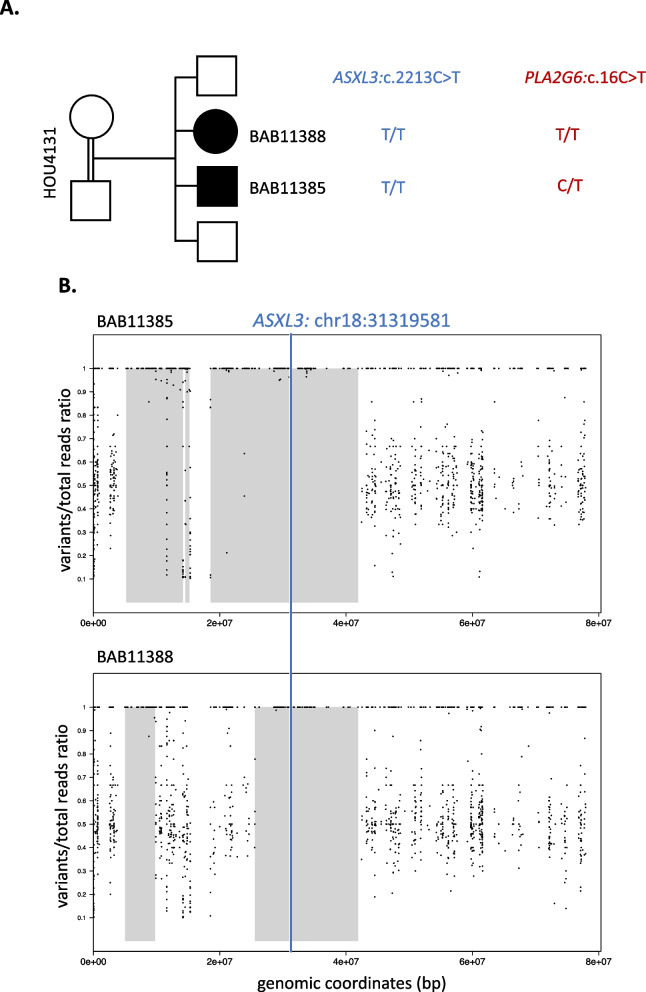


The authors indicate that these errors do not affect any of the results nor the conclusions presented in the text of the study.

The Original Article has been corrected.
