# Supplementary material for: Multilocus pathogenic variants contribute to intrafamilial clinical heterogeneity: a retrospective study of sibling pairs with neurodevelopmental disorders
Source: BMC Med Genomics. 2024 Apr 16;17:85. doi: 10.1186/s12920-024-01852-4 (PMC11020671; doi:10.1186/s12920-024-01852-4)
Supplement: Supplementary file 1 — Additional file 1: Supplementary Fig. 1. Overview of the study. Flow chart depicting study design. We performed a retrospective analysis of 47 sibling pairs diagnosed with neurodevelopmental disorders (NDDs) within the Baylor Hopkins Center for Mendelian Genomics (BHCMG) cohort. The variants responsible for their phenotypes were previously documented by Karaca et al. (n = 24) [17] and Mitani et al. (n = 23) [4]. [file 12920_2024_1852_MOESM1_ESM.pdf]

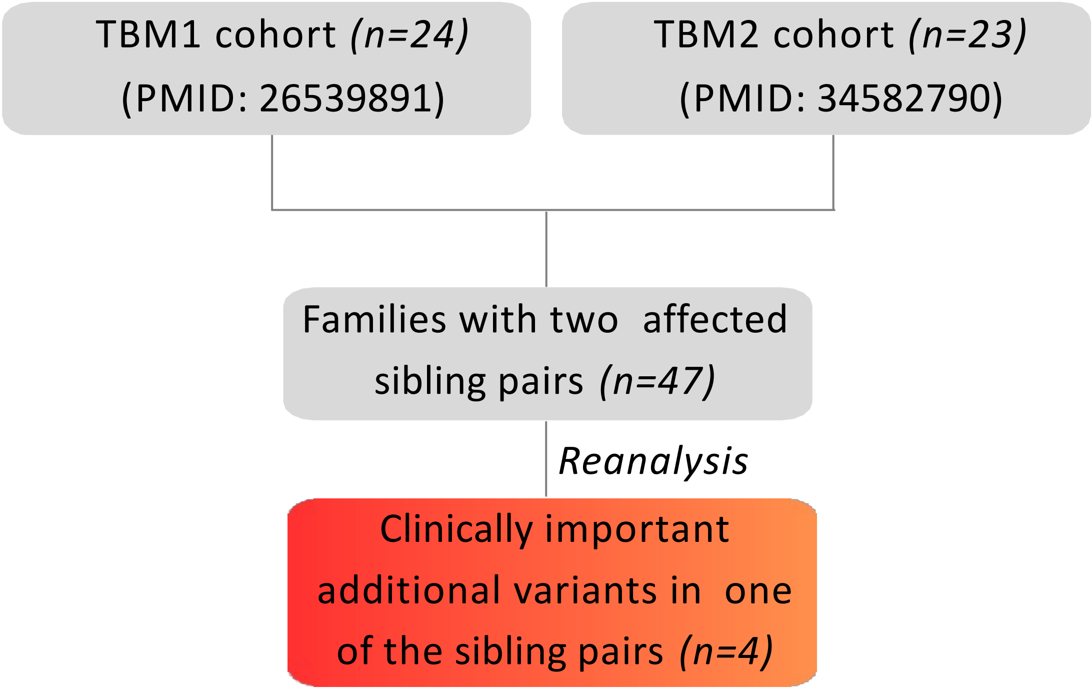
TBM1 cohort *(n=24)*
(PMID: 26539891)
TBM2 cohort *(n=23)*
(PMID: 34582790)
Families with two affected sibling pairs *(n=47)*
Reanalysis
Clinically important additional variants in one of the sibling pairs *(n=4)*
