# Supplementary figures and images for: Multilocus pathogenic variants contribute to intrafamilial clinical heterogeneity: a retrospective study of sibling pairs with neurodevelopmental disorders
Source: BMC Med Genomics. 2024 Apr 16;17:85. doi: 10.1186/s12920-024-01852-4 (PMC11020671; doi:10.1186/s12920-024-01852-4)

A.

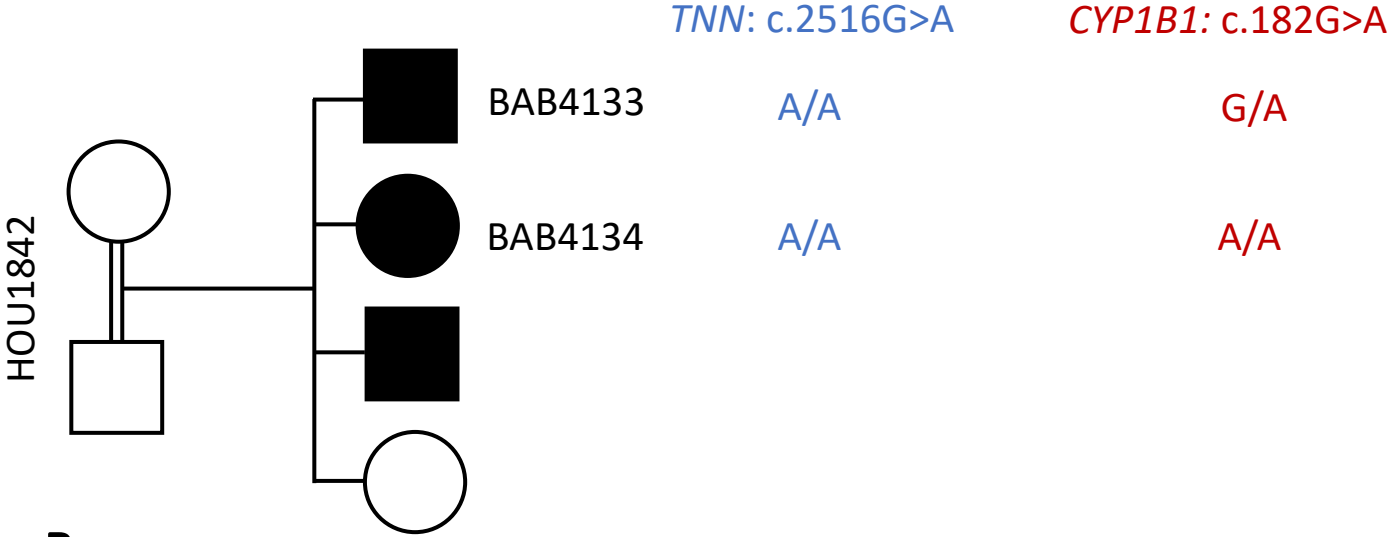

B.

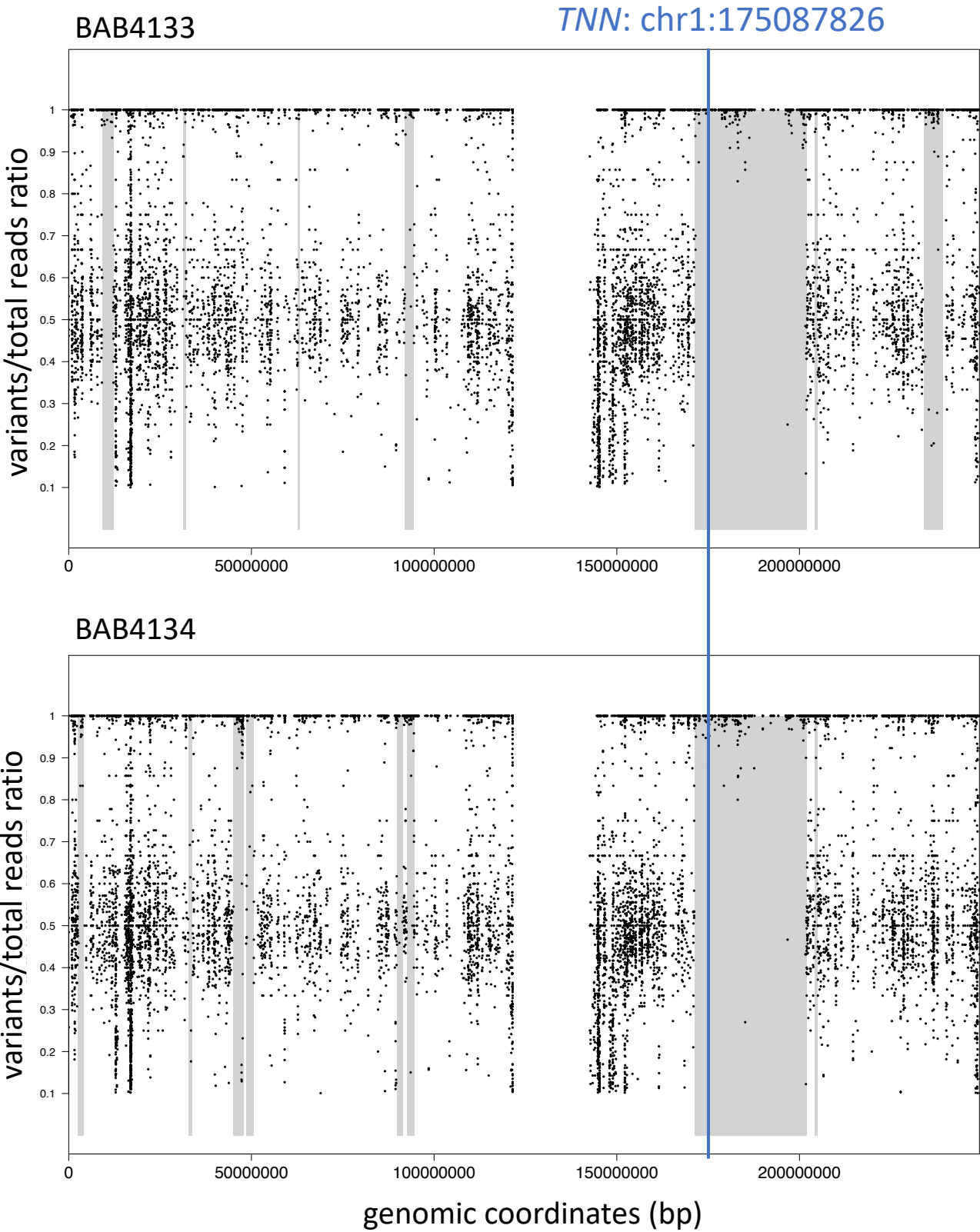

Supplement: Supplementary file 2 — Additional file 2: Supplementary Fig. 2. Pedigree and ROH plots of family HOU1842. A) Pedigree structure and molecular findings in HOU1842. B) B-allele frequency calculated from ES data is visualized as horizontal black dots. ROH blocks are marked by gray rectangles. The shared TNN variant in BAB4133 and BAB4134 is in an ROH region; the blue vertical line marks the position of the variant allele. [file 12920_2024_1852_MOESM2_ESM.pdf]

A.

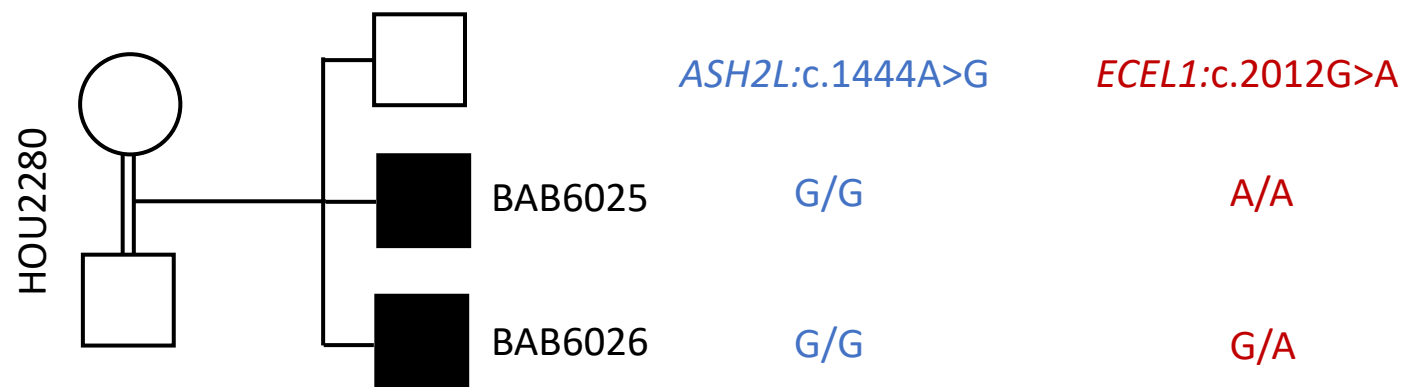

B.

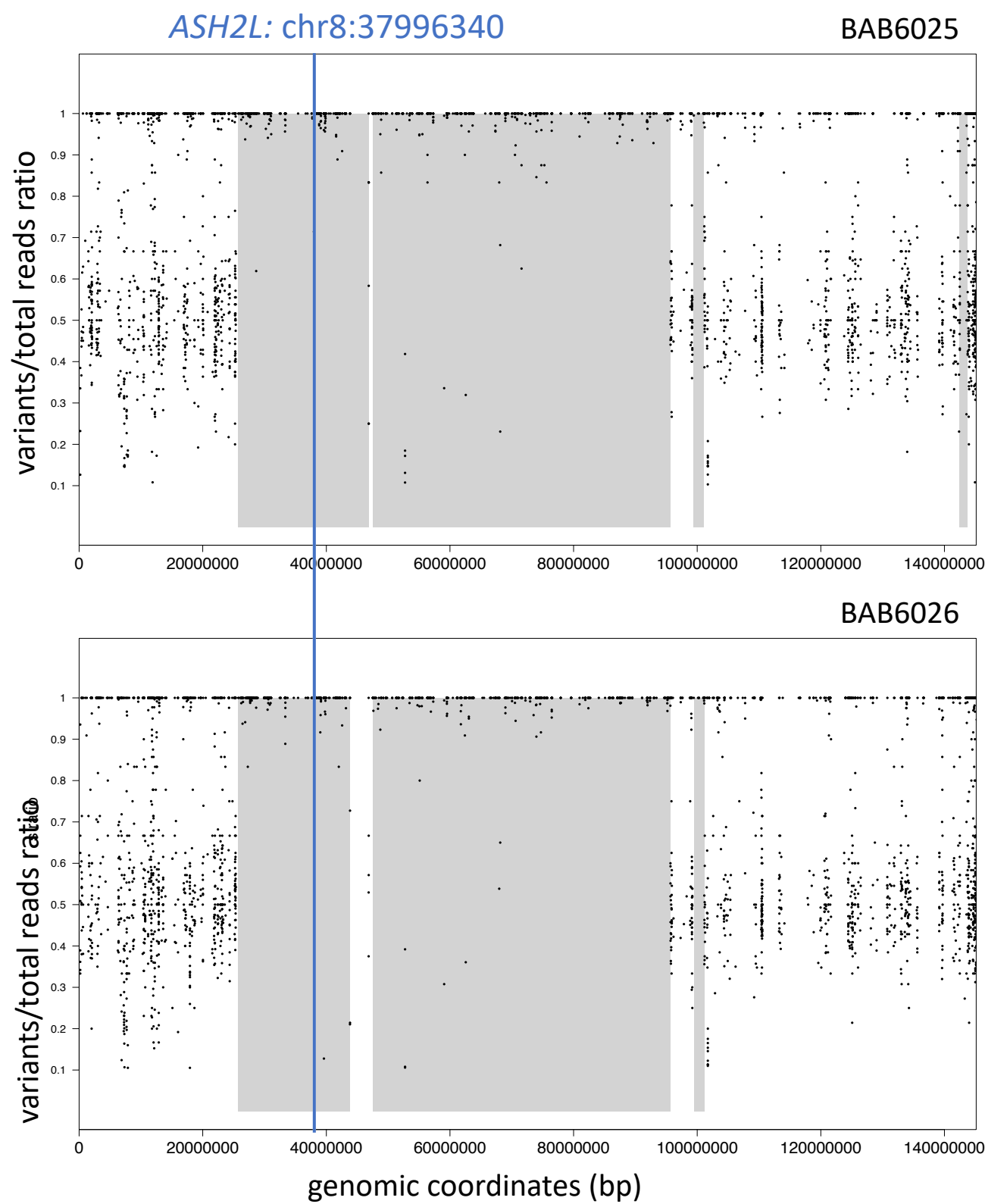

Supplement: Supplementary file 3 — Additional file 3: Supplementary Fig. 3. Pedigree and ROH plots of family HOU2280. A) Pedigree structure and molecular findings in HOU2280. B) B-allele frequency calculated from ES data is visualized as horizontal black dots. ROH blocks are marked by gray rectangles. The shared ASH2L variant in BAB6025 and BAB6026 is in an ROH region; the blue vertical line marks map the position of the variant allele. [file 12920_2024_1852_MOESM3_ESM.pdf]

A.

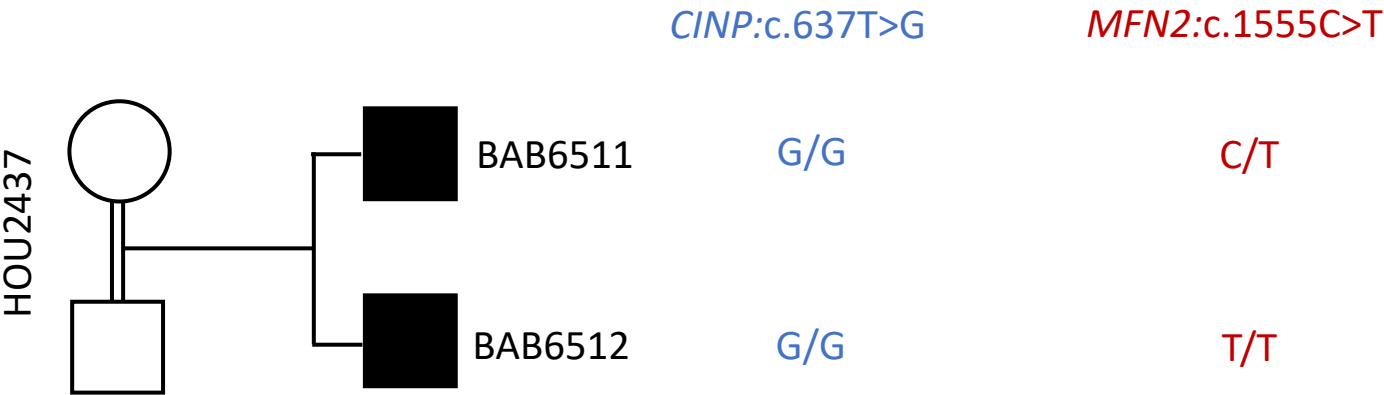

B.

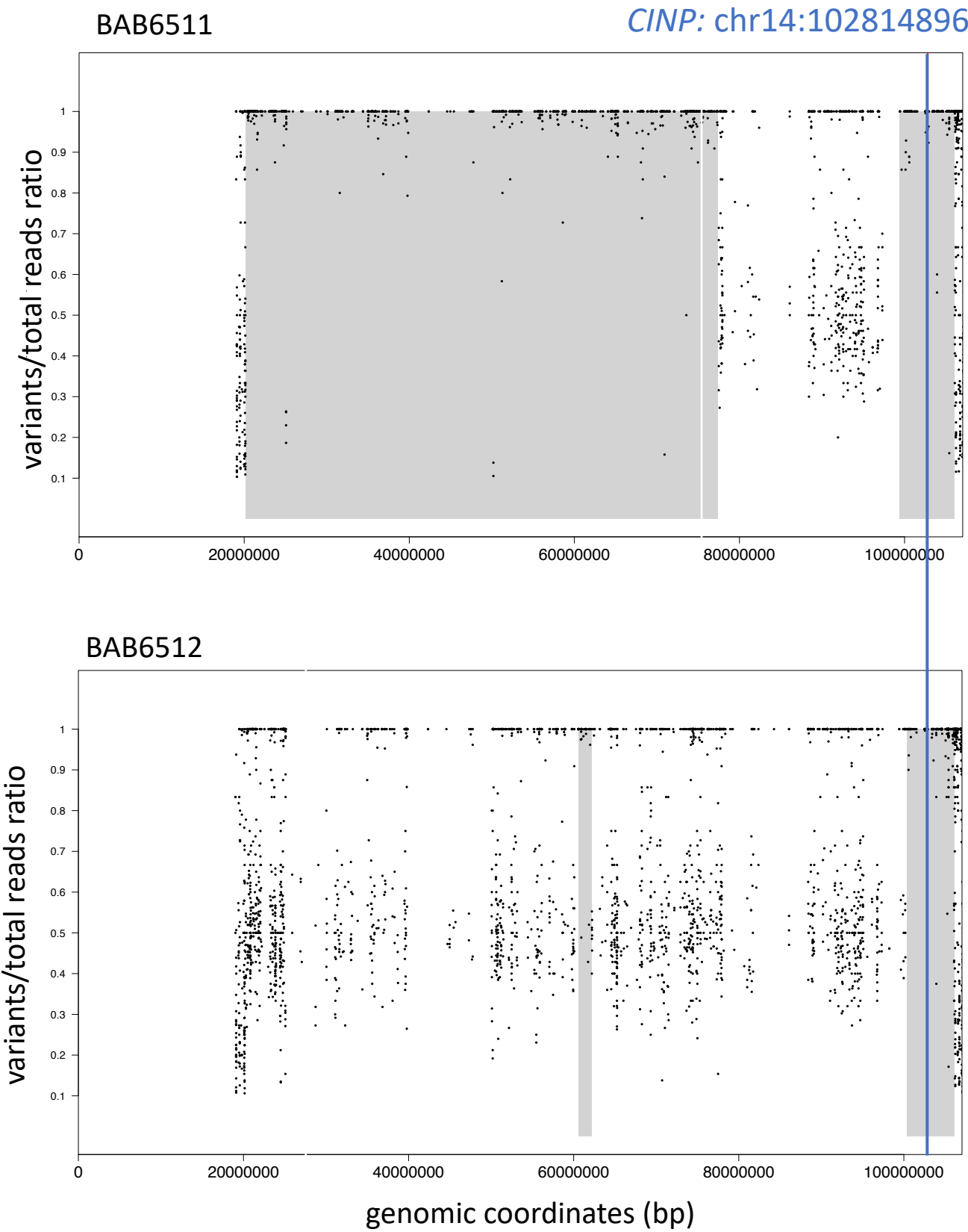

Supplement: Supplementary file 4 — Additional file 4: Supplementary Fig. 4. Pedigree and ROH plots of family HOU2437. A) Pedigree structure and molecular findings in HOU2437. B) B-allele frequency calculated from ES data is visualized as horizontal black dots. ROH blocks are marked by gray rectangles. The shared CINP variant in BAB6511 and BAB6512 is in an ROH region; the blue vertical line marks map the position of the variant allele. [file 12920_2024_1852_MOESM4_ESM.pdf]

A.

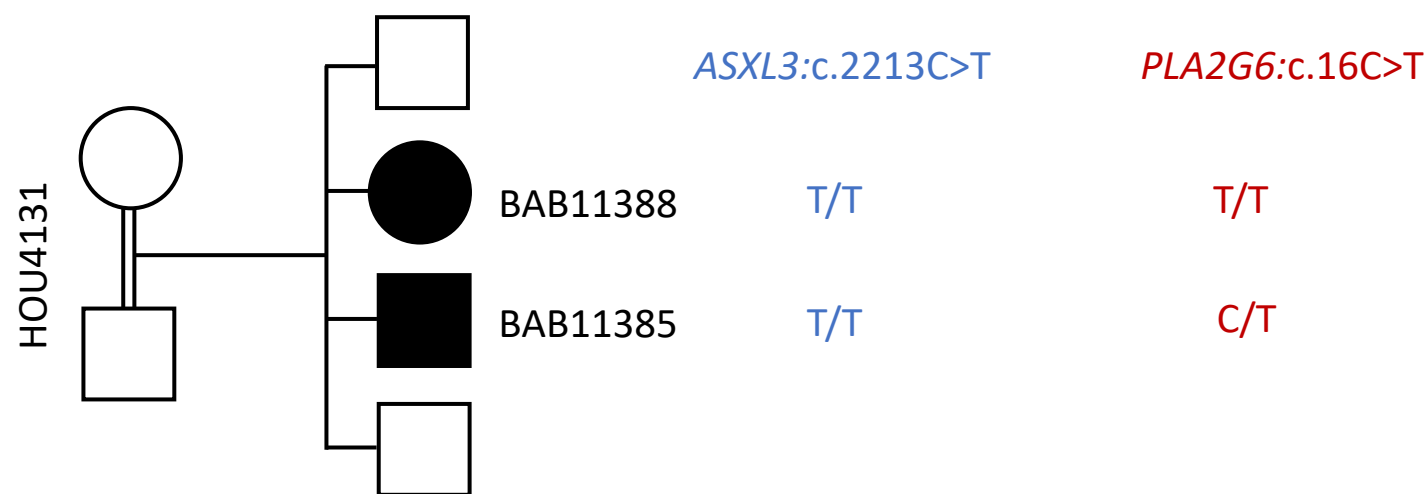

B.

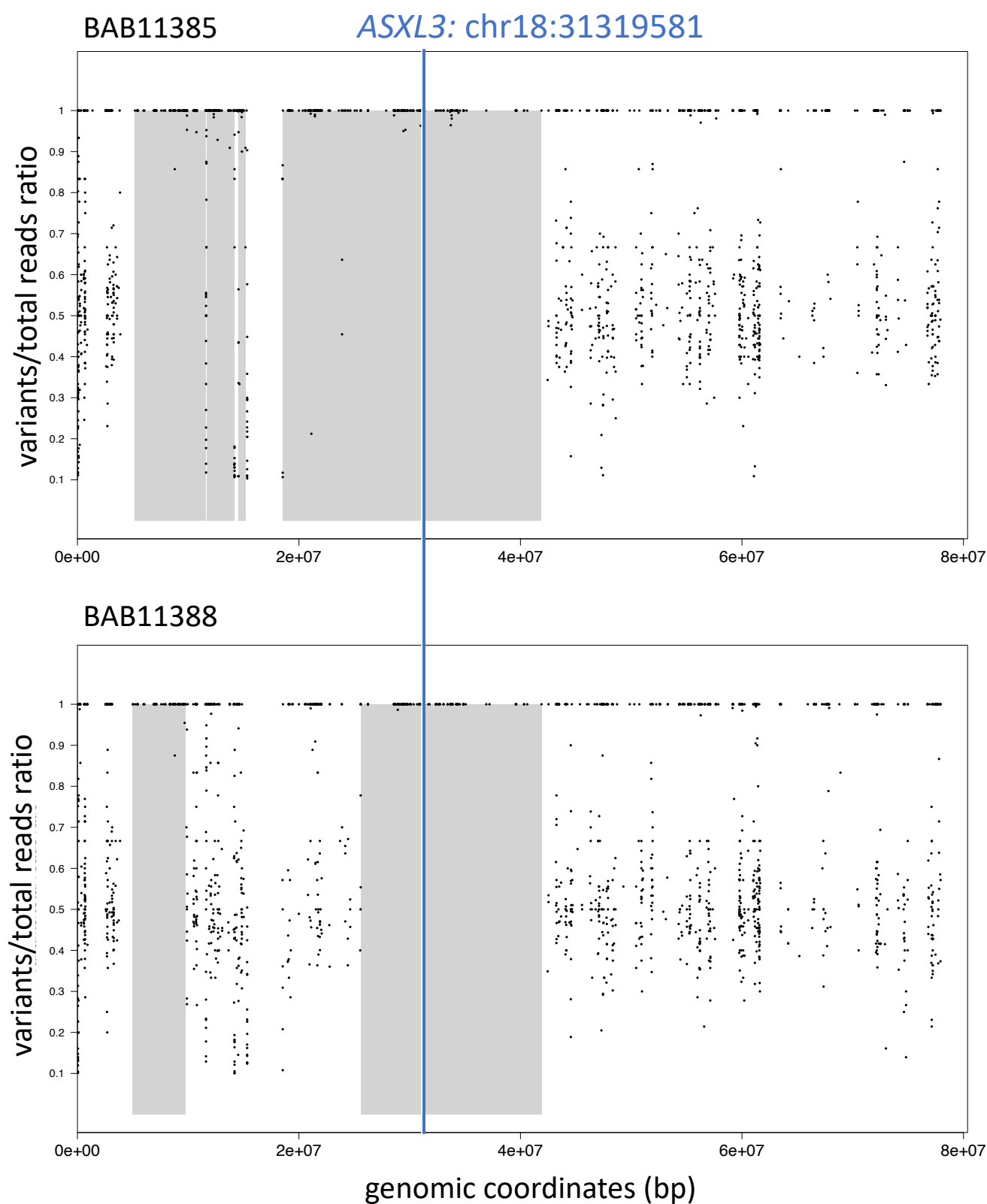

Supplement: Supplementary file 5 — Additional file 5: Supplementary Fig. 5. Pedigree and ROH plots of family HOU4131. A) Pedigree structure and molecular findings in HOU4131. B) B-allele frequency calculated from ES data is visualized as horizontal black dots. ROH blocks are marked by gray rectangles. The shared ASXL3 variant in BAB11385 and BAB11388 is within an ROH region; the blue vertical line marks map the position of the variant allele. [file 12920_2024_1852_MOESM5_ESM.pdf]

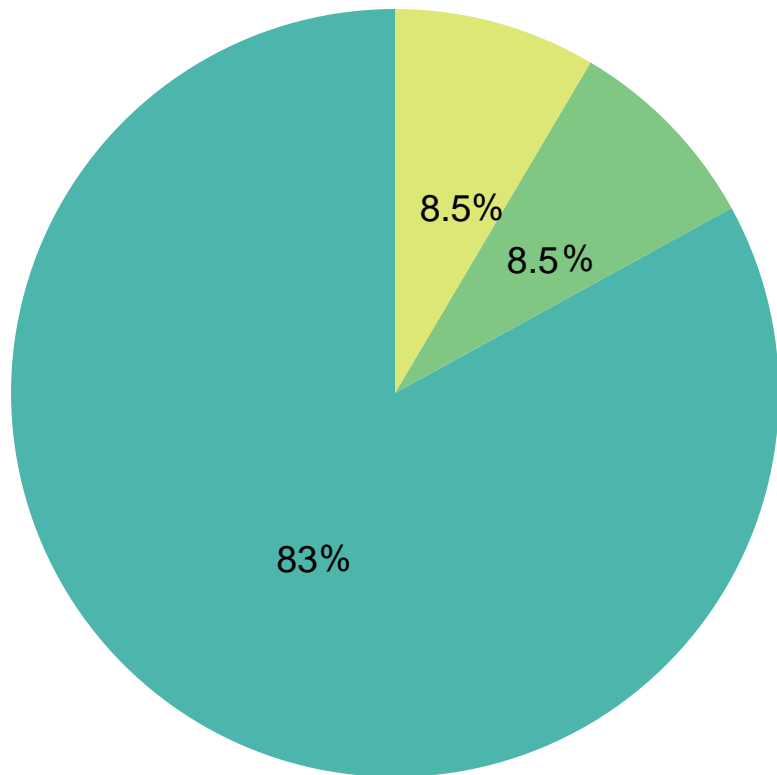

ROH\_pattern

Long

Medium

Non-ROH

Supplement: Supplementary file 6 — Additional file 6: Supplementary Fig. 6. Percentage of ROH size categories covering previously identified pathogenic variants in the patients. 83% of previously detected variants are within a long ROH (light blue); whereas 8.5% of them reside in a medium ROH (light green) and non-ROH (light yellow). It is noteworthy that 76.6% of the sibling pairs (36/47) were reported to be born to a consanguineous family by historical report. [file 12920_2024_1852_MOESM6_ESM.pdf]

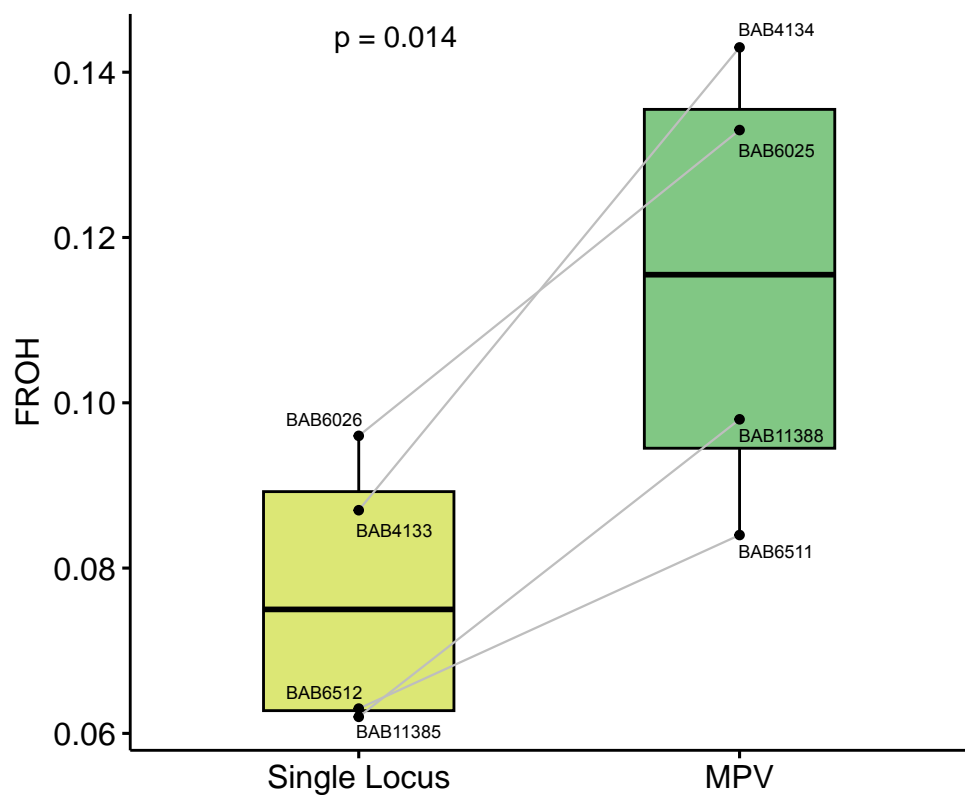

Supplement: Supplementary file 7 — Additional file 7: Supplementary Fig. 7. Box plot of FROH in siblings with MPV vs. siblings with single locus pathogenic variant. The average FROH values are notably higher in siblings with multiple pathogenic variants (MPVs) when compared to those with a single locus variant (p-value = 1.4e-2). The gray lines connect the FROH value data points for each sibling pair. [file 12920_2024_1852_MOESM7_ESM.pdf]
