# Supplementary material for: Multilocus pathogenic variants contribute to intrafamilial clinical heterogeneity: a retrospective study of sibling pairs with neurodevelopmental disorders
Source: BMC Med Genomics. 2024 Apr 16;17:85. doi: 10.1186/s12920-024-01852-4 (PMC11020671; doi:10.1186/s12920-024-01852-4)
Supplement: Supplementary file 8 — Additional file 8: Supplementary Table 1. The total size of short, medium, and long ROH regions and FROH values in each individual. [file 12920_2024_1852_MOESM8_ESM.docx]

| Family IDs | Individual IDs | Variant Status | Short ROH (Mb) | Medium ROH (Mb) | Long ROH (Mb) | F_ROH_ |
| --- | --- | --- | --- | --- | --- | --- |
| HOU1842 | BAB4133 | Single locus | 32.17 | 35.74 | 238.16 | 0.087 |
|  | BAB4134 | MPV | 29.96 | 61.64 | 379.11 | 0.143 |
| HOU2280 | BAB6025 | MPV | 28.1 | 54.41 | 366.3 | 0.133 |
|  | BAB6026 | Single locus | 34.3 | 57.5 | 247.89 | 0.096 |
| HOU2437 | BAB6511 | MPV | 23.48 | 38.13 | 232.16 | 0.084 |
|  | BAB6512 | Single locus | 31.15 | 40.5 | 161.89 | 0.063 |
| HOU4131 | BAB11385 | Single locus | 39.14 | 60.78 | 159.52 | 0.062 |
|  | BAB11388 | MPV | 35.63 | 60.5 | 248.03 | 0.098 |
