# Supplementary material for: Multilocus pathogenic variants contribute to intrafamilial clinical heterogeneity: a retrospective study of sibling pairs with neurodevelopmental disorders
Source: BMC Med Genomics. 2024 Apr 16;17:85. doi: 10.1186/s12920-024-01852-4 (PMC11020671; doi:10.1186/s12920-024-01852-4)
Supplement: Supplementary file 9 — Additional file 9: Supplementary file. The primer sequences used in the orthogonal confirmation. [file 12920_2024_1852_MOESM9_ESM.docx]

PLA2G6

F: TATGTTCCCGCTGAGCATC

R: GCCTGAGGGTTCTGTCACTT

MFN2

F: ATCCCTGGCAGTAGCTGGTA

R: TCCAGGTCAATCAGTAGCAAAA

ECEL1

F: GCTCTGTCCCTCATCTGGAG

R: TGTCCAAACAAAACCTGTGC

CYP1B1

F: CCTTCCAGTGCTCCGAGTAG

R: GTCTCTGCACCCCTGAGTGT
